# Population‐based socio‐demographic platforms as a critical tool for contextual and equitable ADRD research in rapidly ageing African societies

**DOI:** 10.1002/alz70860_102700

**Published:** 2025-12-23

**Authors:** Stephen Tollman, HAALSI research team, USA and South Africa

**Affiliations:** ^1^ University of the Witwatersrand, Johannesburg, xx, South Africa

## Abstract

Rapid social, population and health transitions characterize much of rural Africa today. Integral to the complexity of change are rising life expectancy coupled with premature onset of multimorbidity, coupling infectious and non‐communicable conditions.

Understanding and awareness of Alzheimer's Disease and Related Dementias (ADRD) in such contexts is weak to non‐existent. In consequence, scientific and policy communities, and the public at large, risk falling far behind the curve of evidence needed to mount effective, community‐sensitive responses.

Investment in population‐based cohorts including health and socio‐demographic surveillance platforms are critical instruments in these environments. Such capability is needed to generate the necessary data, support the required analyses, provide foundations for intervention‐evaluation, and contribute to public understanding.

This contribution to a session on Perspectives will draw on data and analyses from 10‐plus years of the HAALSI cohort: Health and Aging in Africa: longitudinal studies in South Africa which is based in the country's rural northeast adjacent to southern Mozambique. HAALSI is nested within the long‐running Agincourt health and socio‐demographic surveillance system (HDSS) – a whole population cohort of ∼120,000 people in 22,000 households that together make up 31 village communities.

The breadth of findings from this community‐representative cohort will be shared – providing an acute insight into dementia prevalence, cognitive trajectories, behavioral and social drivers of positive/negative cognitive change, mortality transition including cause‐of‐death, and community responses.

In this rapidly transitioning, high HIV context with escalating cardiometabolic risk, the scope for preventive and promotive responses is high with potential for marked impact on dementias, their cognitive antecedents, and underlying clinical, behavioral and social risk factors manifesting along the life course.

Altogether, the HAALSI experience is an exemplar of the contributions possible by capitalizing on longitudinal population‐based platforms responsive to communities and other stakeholders. Such in‐depth, community representative platforms can provide invaluable testbeds for the questionnaire sets, screening tests, sample collection, field research methods, and community engagement necessary to

